# Developing and validating nomograms for predicting the survival in patients with clinical local-advanced gastric cancer

**DOI:** 10.3389/fonc.2022.1039498

**Published:** 2022-10-26

**Authors:** Chong Hou, Fangxu Yin, Yipin Liu

**Affiliations:** ^1^ Department of Gastroenterology, Yantai Affiliated Hospital of Binzhou Medical University, Yantai, China; ^2^ Department of Thyroid and Breast Surgery, Binzhou Medical University Hospital, Binzhou, China

**Keywords:** locally advanced gastric cancer, overall survival, prognosis, cancer specific survival, SEER, nomogram

## Abstract

**Background:**

Many patients with gastric cancer are at a locally advanced stage during initial diagnosis. TNM staging is inaccurate in predicting survival. This study aims to develop two more accurate survival prediction models for patients with locally advanced gastric cancer (LAGC) and guide clinical decision-making.

**Methods:**

We recruited 2794 patients diagnosed with LAGC (2010–2015) from the Surveillance, Epidemiology, and End Results (SEER) database and performed external validation using data from 115 patients with LAGC at Yantai Affiliated Hospital of Binzhou Medical University. Univariate and multifactorial survival analyses were screened for meaningful independent prognostic factors and were used to build survival prediction models. Concordance index (C-index), receiver operating characteristic (ROC) curves, calibration curves, and decision curve analysis (DCA) were evaluated for nomograms. Finally, the differences and relationships of survival and prognosis between the three different risk groups were described using the Kaplan–Meier method.

**Results:**

Cox proportional risk regression model analysis identified independent prognostic factors for patients with LAGC, and variables associated with overall survival (OS) included age, race, marital status, T-stage, N-stage, grade, histologic type, surgery, and chemotherapy. Variables associated with cancer-specific survival (CSS) included age, race, T-stage, N-stage, grade, histological type, surgery, and chemotherapy. In the training cohort, C-index of nomogram for predicting OS was 0.722 (95% confidence interval [95% CI]: 0.708–0.736] and CSS was 0.728 (95% CI: 0.713–0.743). In the external validation cohort, C-index of nomogram for predicted OS was 0.728 (95% CI:0.672–0.784) and CSS was 0.727 (95% CI:0.668–0.786). The calibration curves showed good concordance between the predicted and actual results. C-index, ROC, and DCA results indicated that our nomograms could more accurately predict OS and CSS than TNM staging and had a higher clinical benefit. Finally, to facilitate clinical use, we set up two web servers based on nomograms.

**Conclusion:**

The nomograms established in this study have better risk assessment ability than the clinical staging system, which can help clinicians predict the individual survival of LAGC patients more accurately and thus develop appropriate treatment strategies.

## Introduction

Gastric cancer is one of the most common malignancies worldwide. According to GLOBOCAN 2018, it ranks fifth in incidence and third in overall mortality among all cancers, after lung and colorectal cancers (1). Among the East Asian population, gastric cancer has the highest mortality rate, and about 42% of gastric cancer patients worldwide are in China (2). Because early gastric cancer is not clinically evident, most gastric cancer patients are at locally advanced stages when diagnosed clinically (1, 3, 4). Surgery is currently the primary treatment modality for locally advanced gastric cancer (LAGC) (5). Since 2000, adjuvant treatments have also been found to be effective in LAGC patients (6). Preoperative neoadjuvant chemotherapy has garnered widespread attention in recent years, but whether LAGC patients can achieve a good survival benefit from it remains controversial and lacks a consensus. Recently, the treatment paradigm for LAGC has shifted from single surgery to a multidisciplinary and comprehensive treatment based on surgery (4). Although many treatment options exist, relevant studies have revealed that the five-year survival rate of treated gastric cancer patients is less than 25%, and most treated gastric cancer patients will have a recurrence within 2 or 5 years (7–13).

The American Joint Committee on Cancer (AJCC) staging system is a common tool used clinically to predict disease progression and design treatment strategies (14, 15). However, the clinical staging system relies only on anatomical and pathological features for disease assessment, and many other important prognostic factors, such as age, gender, and race, are not considered (16, 17). Numerous studies have revealed that clinical staging alone is insufficient to predict the prognosis of cancer patients (17–20). Therefore, there is a need to establish a more accurate prognostic assessment protocol.

Nomogram is a reliable and convenient prognostic prediction tool. It can quantitatively predict the prognosis of each cancer patient based on multiple prognostic factors to provide more effective individualized medical treatment, and it is widely recognized and used in the international arena (21, 22). For clinicians, the prognostic stratification of LAGC remains a clinical challenge to be solved. The development of individualized treatment strategies has received much attention internationally as one of the major clinical challenges in recent years. However, studies on the development and validation of LAGC nomograms have not been comprehensively sufficient. In this regard, we constructed and validated two reliable nomograms to predict overall survival (OS) and cancer-specific survival (CSS) in LAGC patients, thus helping clinicians to more accurately assess patients’ prognoses and develop more individualized treatment plans.

## Materials and methods

### Data sources and patient selection

Our study was based on data information from the Surveillance, Epidemiology, and End Results (SEER) database and Yantai Affiliated Hospital of Binzhou Medical University (YAHBMU) for LAGC patients. The SEER database is a National Cancer Institute (NCI)-funded project covering approximately 30% of the U.S. population and contains data information from 18 cancer registries with large sample sizes, wide population coverage, high data accuracy, and multicenter and multi-regional registries of case samples advantages (23).

We collected data information on 2794 (2010–2015) LAGC patients from SEER database. Inclusion criteria were as follows: (i) gastric cancer patients with clinical stage T1-2N+M0 and T3-4N0/+M0; (ii) patients with no other confirmed tumors other than LAGC; (iii) complete clinical and pathological data; (iv) complete follow-up information. Exclusion criteria were as follows: (i) patient age < 18 years at diagnosis; (ii) unknown data on race, grade, surgery and marital status; and (iii) follow-up time of 0 and unknown. We assigned approximately 70% of these LAGC patients (n = 1,946) to the training group, and the remaining 30% (n = 848) to the internal validation group using R software. If the sample size of the missing value in the database was less than 5% of the total number of people, it will be deleted. We also collected data information of 115 (2014–2018) LAGC patients from YAHBMU, which served as an external validation cohort. Inclusion and exclusion criteria were as above. Follow-up visits were conducted through direct contact with patients or telephone conversations. The follow-up ended on June 30, 2022. This study was approved by the YAHBMU Ethics Committee.

### Construction and validation of the nomogram

This study was designed on the transparent reporting of a multivariable prediction model for individual prognosis or diagnosis (TRIPOD) (24). The endpoints of this study were OS and CSS in patients with LAGC. First, we obtained the following variables from the SEER database: age at diagnosis, race, gender, marital status, grade, T-stage, N-stage, histological type, surgery, radiotherapy, and chemotherapy. Significant variables were screened out using univariate COX analysis, followed by multivariate analysis, and independent and significant prognostic factors associated with LAGC were screened out by multivariate analysis after excluding confounding factors. The independent prognostic factors were integrated into the nomogram, and the scores of each independent prognostic factor were summed by transformation to obtain the OS and CSS of LAGC patients at 1, 3, and 5 years. In the validation, we used the concordance index (C-index) to assess the accuracy of the nomogram prediction and receiver operating characteristic (ROC) curves to evaluate the sensitivity and specificity of the nomogram. Furthermore, we used calibration curves to compare the predicted and actual results of nomograms. Finally, we used decision curve analysis (DCA) to evaluate the potential value of the nomogram.

### Risk stratification based on the nomogram

We used the X-tile software (version 3.6.1; Robert, MD) to divide the ages into three groups in the training set. The scores of each independent prognostic factor were summed to obtain the total risk score of LAGC patients. Using the best cut-off value of the total risk score determined by X-Tile software (version 3.6.1), LAGC patients were classified into high-, intermediate-, and low-risk groups. Kaplan–Meier curve was employed to assess OS and CSS of LAGC patients.

### Statistical analysis

The obtained data were statistically analyzed using SPSS software version 22.0 and R software version 4.1.2. The data were analyzed by the χ2 test or Fisher’s exact probability method. The Kaplan–Meier method was utilized to plot the survival curves, and the log-rank test was used to compare the survival rates. After setting the seed number, LAGC patients were randomly divided into training and internal validation groups in the ratio of 7:3 using the “caret” package in R software. The training cohort was used to determine the independent prognostic factors of LAGC patients and create prognostic nomograms, while internal and external validation cohorts were used to test the accuracy of the nomogram. Univariate and multifactorial COX regression analyses were employed to identify independent prognostic factors for LAGC. After calculating the variance inflation factor (VIF), the VIF values of each covariate were less than 4, suggesting that the multicollinearity between variables is not significant. Based on univariate and multifactorial regression analyses, clinical and pathological characteristics that could be used as independent prognostic factors were included, and “rms,” “foreign,” and “survival” packages of R software were used to construct nomograms of LAGC patients. The degree of differentiation was the ability to distinguish the proposed model from conventional AJCC staging and was measured in terms of C-index and the area under the receiver operator characteristics curve (AUC). The C-index and AUC range from 0.5 to 1. The closer the C-index and AUC values were to 1, the better the model’s differentiation was. A calibration curve was also used to measure the closeness of the predicted risk to the actual risk. The vertical coordinate of the curve was the actual survival rate of LAGC patients, and the horizontal coordinate was the survival rate predicted by the nomogram. By observing the degree of deviation of the curve from the diagonal line, it was possible to determine whether the constructed prediction model could accurately predict OS and CSS at 1, 3, and 5 years. Finally, DCA was used to verify the clinical validity. The difference was indicated as statistically significant at p < 0.05.

## Results

### Data sources and patient selection

**Figure 1:**
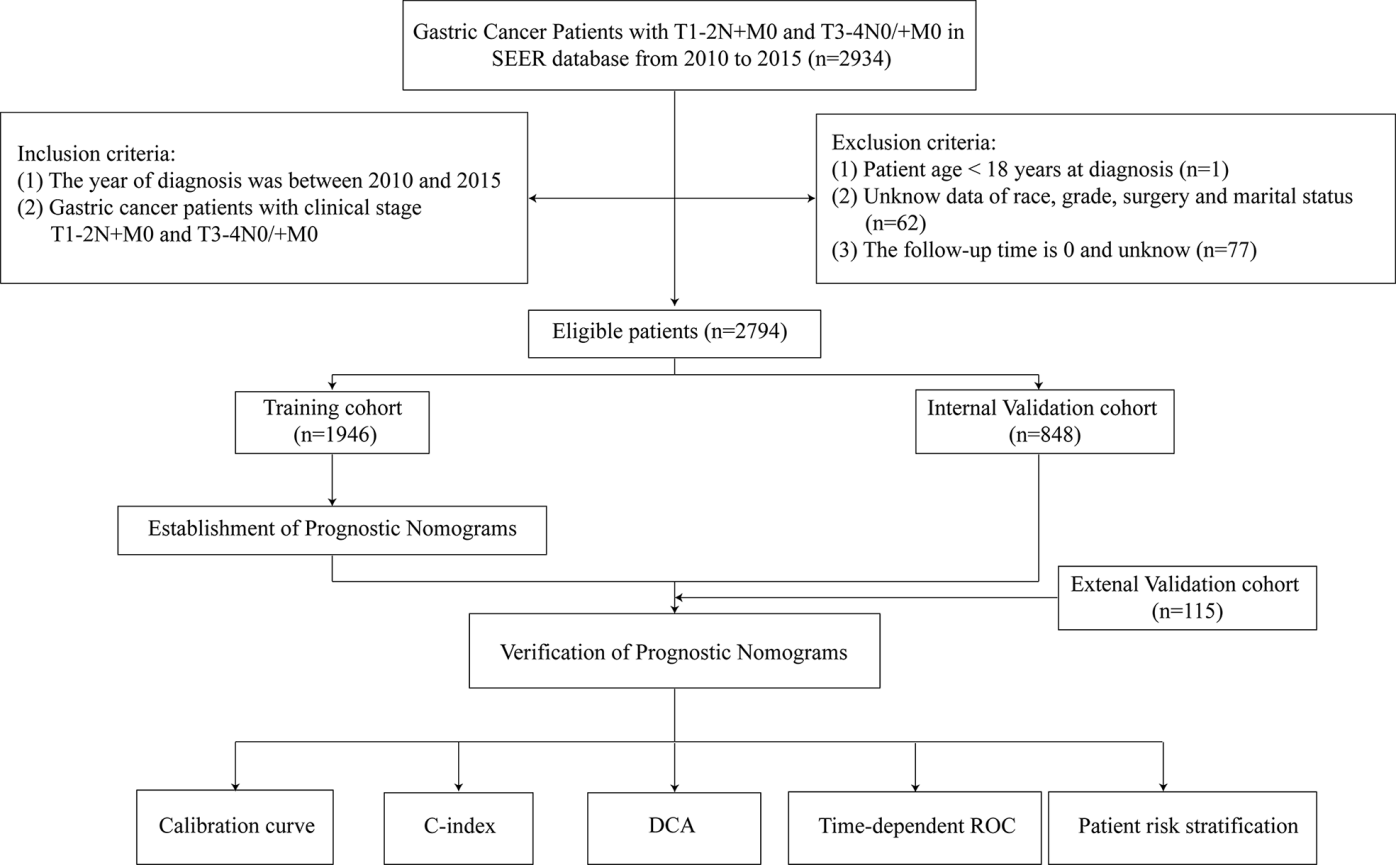
Flowchart of participant inclusion and exclusion.

This study collected information on 2794 eligible LAGC patients (1946 in the training cohort and 848 in the internal validation cohort) from SEER database and external validation of 115 eligible LAGC patients from YAHBMU. Screening details and demographic characteristics are shown in **Figure 1** and **Table 1**, respectively. No statistical differences were observed between the training cohort and the internal validation cohorts (p > 0.05).

**Table 1:** Characteristics of patients with LAGC in the training and validation cohort.

Characteristics	Overall	Training cohort	Validation cohort	External validation cohort	T vs IV	T vs EV
	(n =2794)	(n = 1946)	(n = 848)	(n = 115)	P	P
	No. of patients (%)	No. of patients (%)	No. of patients (%)	No. of patients (%)		
**Age**					0.184	0.369
18-66	1338 (47.9)	928 (47.7)	410 (48.4)	50 (43.5)		
67-80	1032 (36.9)	736 (37.8)	296 (34.9)	43 (37.4)		
81-100	424 (15.2)	282 (14.5)	142 (16.7)	22 (19.1)		
**Race**					0.497	<0.01
White	1855 (66.4)	1302 (66.9)	553 (65.2)	0 (0.0)		
Black	300 (10.7)	211 (10.8)	89 (10.5)	0 (0.0)		
Others^1^	639 (22.9)	433 (22.3)	206 (24.3)	115 (100.0)		
**Sex**					0.387	0.499
Female	943 (33.8)	653 (33.6)	290 (34.2)	38 (33.0)		
Male	1851 (66.2)	1293 (66.4)	558 (65.8)	67 (67.0)		
**Marital status**					0.231	0.476
Married	1753 (62.7)	1223 (62.8)	530 (62.5)	71 (61.7)		
Single	407 (14.6)	295 (15.2)	112 (13.2)	14 (12.2)		
Others^2^	634 (22.7)	428 (22.0)	206 (24.3)	30 (26.1)		
**Grade**					0.063	0.121
I	163 (5.9)	106 (5.4)	57 (6.7)	9 (7.8)		
II	717 (25.6)	477 (24.5)	239 (28.3)	35 (30.5)		
III	1839 (65.8)	1315 (67.6)	524 (61.8)	66 (57.4)		
IV	75 (2.7)	48 (2.5)	27 (3.2)	5 (4.3)		
**AJCC T**					0.846	0.910
T1	184 (6.6)	126 (6.5)	58 (6.8)	8 (7.0)		
T2	269 (9.6)	184 (9.5)	85 (10.0)	11 (9.6)		
T3	1567 (56.1)	1102 (56.6)	465 (54.9)	68 (59.1)		
T4	774 (27.7)	534 (27.4)	240 (28.3)	28 (24.3)		
**AJCC N**					0.719	0.577
N0	765 (27.4)	537 (27.6)	228 (26.9)	26 (22.6)		
N1	1056 (37.8)	735 (37.8)	321 (37.9)	48 (41.7)		
N2	502 (18.0)	340 (17.4)	162 (19.0)	23 (20.0)		
N3	471 (16.8)	334 (17.2)	137 (16.2)	18 (15.7)		
**Surgery**					0.863	0.722
Yes	2149 (76.9)	1495 (76.8)	654 (77.1)	90 (78.3)		
No	645 (23.1)	451 (23.2)	194 (22.9)	25 (21.7)		
**Radiation**					0.387	0.071
Yes	1369 (49.0)	964 (49.5)	405 (47.8)	47 (40.9)		
No/Unknown	1425 (51.0)	982 (50.5)	443 (52.2)	68 (59.1)		
**Chemotherapy**					0.223	0.063
Yes	2082 (74.5)	1463 (75.2)	619 (73.0)	77 (67.0)		
No/Unknown	712 (25.5)	483 (24.8)	229 (27.0)	38 (33.0)		
**Histologic Type**					0.332	0.071
Adenocarcinoma	1879 (67.3)	1321 (67.8)	558 (65.8)	80 (69.6)		
Undifferentiated	8 (0.3)	4 (0.2)	4 (0.5)	2 (1.7)		
SRCC	472 (16.9)	330 (17.0)	142 (16.7)	20 (17.4)		
Special Type	435 (15.5)	291 (15.0)	144 (17.0)	13 (11.3)		

SRCC, signet ring cell carcinoma; HR, hazard ratio; 95 CI, 95% confidence interval; Others¹, including Asian or Pacific Islander and American Indian/Alaska Native; Others², including separated, divorced and widowed; T, Training cohort; IV, Internal validation cohort; EV, External validation cohort.

As listed in **Table 1**, more than half of the patients (52.1%) were older than 66 years, 66.4% were white, 10.7% were black, and 22.9% were of other races. Most patients with LAGC were male, accounting for 66.2%. Also, 62.7% of LAGC patients were married, while 14.6% were unmarried. Patients with grade III were the most in number, accounting for 65.8%, followed by those with grade II, accounting for 25.6%. More than half of the total patients had T3 stage, accounting for 56.1%. Each N-stage was more evenly distributed, with N0 accounting for 27.4%, N1 for 37.8%, N2 for 18.0%, and N3 for 16.8%. The largest number of patients had adenocarcinoma, accounting for 67.3%. In addition, 23.1% of patients did not undergo surgery, 51.0% did not receive radiotherapy, and 25.5% did not receive chemotherapy.

### Development and validation of prognostic nomograms

**Figure 2:**
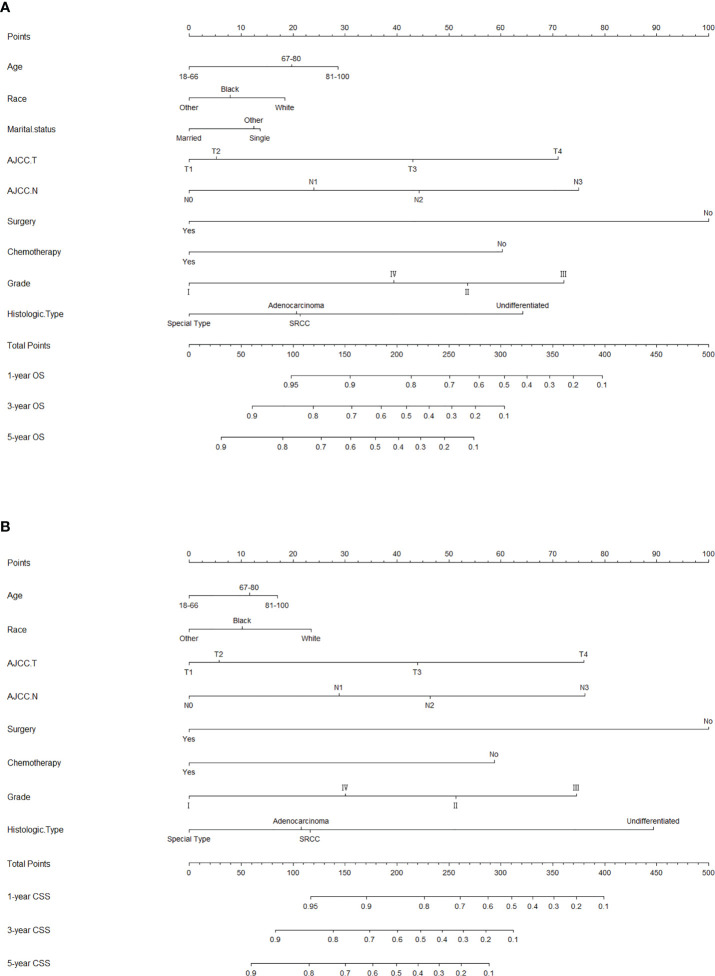
Nomograms for predicting 1-, 3-, and 5-year **(A)** OS and **(B)** CSS of patients with LAGC.

**Figure 3:**
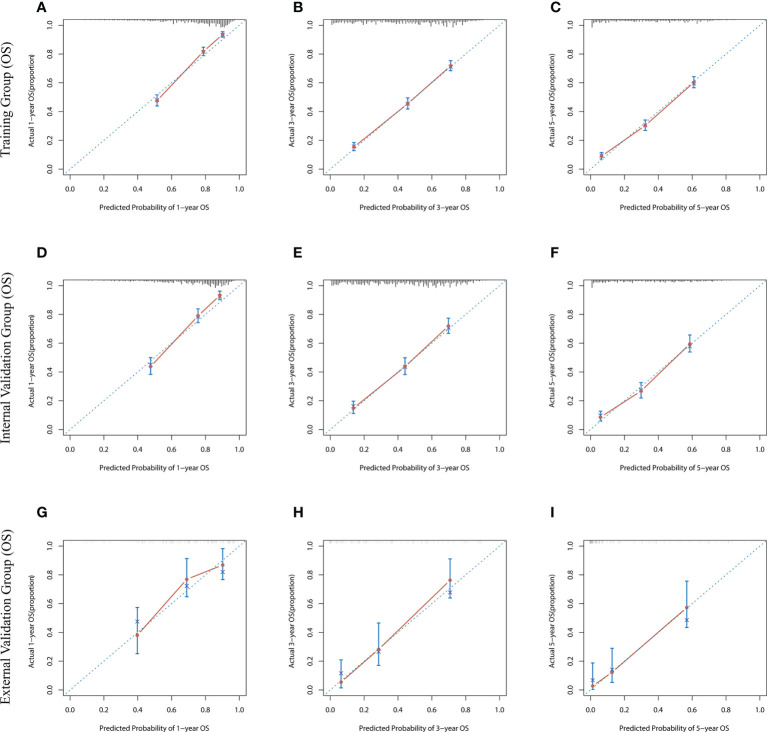
The calibration curves for predicting OS at **(A)** 1-year and **(B)** 3-year and **(C)** 5-year in the training cohort, and at **(D)** 1-year **(E)** 3-year and **(F)** 5-year in the internal validation cohort, and at **(G)** 1-year **(H)** 3-year and **(I)** 5-year in the external validation cohort.

**Figure 4:**
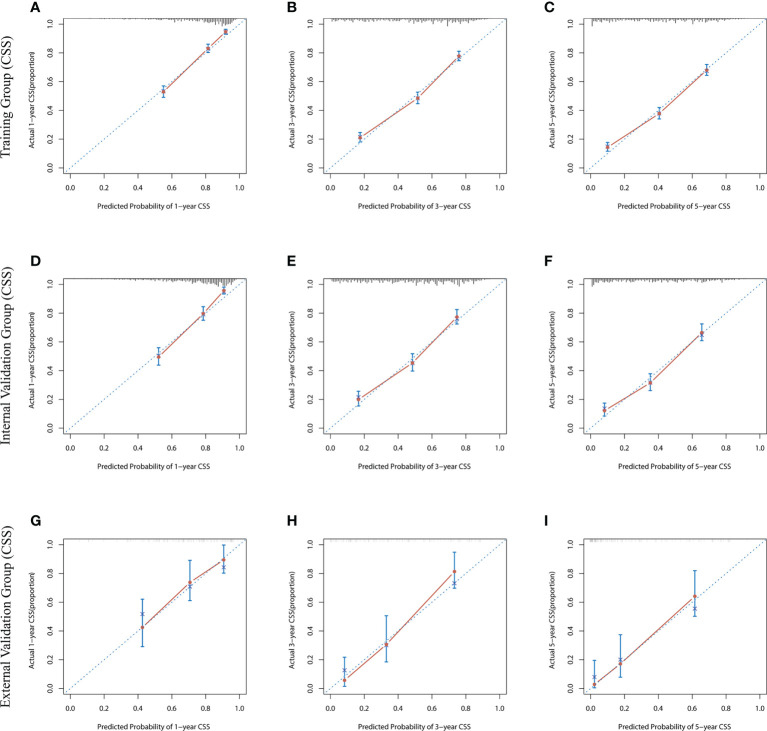
The calibration curves for predicting CSS at **(A)** 1-year and **(B)** 3-year and **(C)** 5-year in the training cohort, and at **(D)** 1-year **(E)** 3-year and **(F)** 5-year in the internal validation cohort, and at **(G)** 1-year **(H)** 3-year and **(I)** 5-year in the external validation cohort.

**Figure 5:**
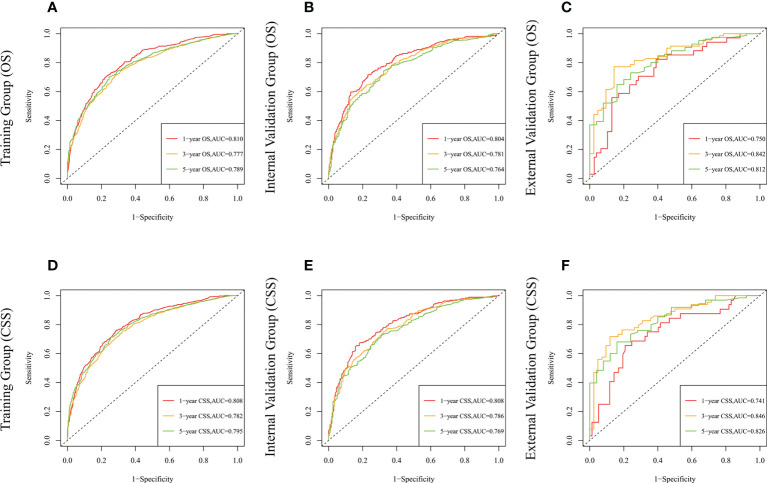
The time-dependent ROC curves of the nomogram predicting OS at **(A)** 1-year and 3-year and 5-year in the training cohort, and at **(B)** 1-year 3-year and 5-year in the internal validation cohort, **(C)** 1-year and 3-year and 5-year in the external training cohort. The time-dependent ROC curves of the nomogram predicting CSS at **(D)** 1-year and 3-year and 5-year in the training cohort, and at **(E)** 1-year 3-year and 5-year in the internal validation cohort, **(F)** 1-year and 3-year and 5-year in the external training cohort.

**Figure 6:**
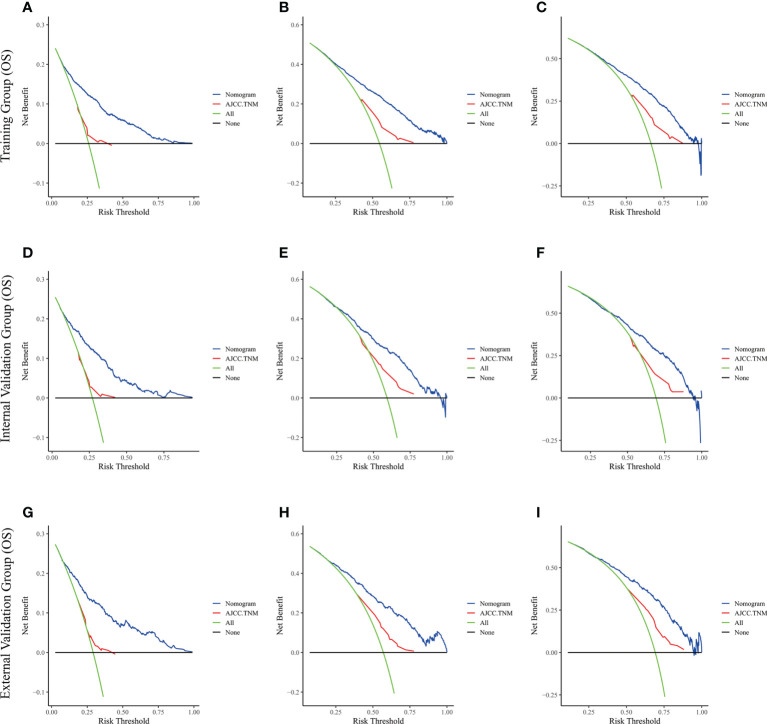
The decision curve analysis of the nomogram and AJCC.TNM for OS at **(A)** 1-year and **(B)** 3-year and **(C)** 5-year in the training cohort, and at **(D)** 1-year **(E)** 3-year and **(F)** 5-year in the internal validation cohort, and at **(G)** 1-year **(H)** 3-year and **(I)** 5-year in the external validation cohort.

**Figure 7:**
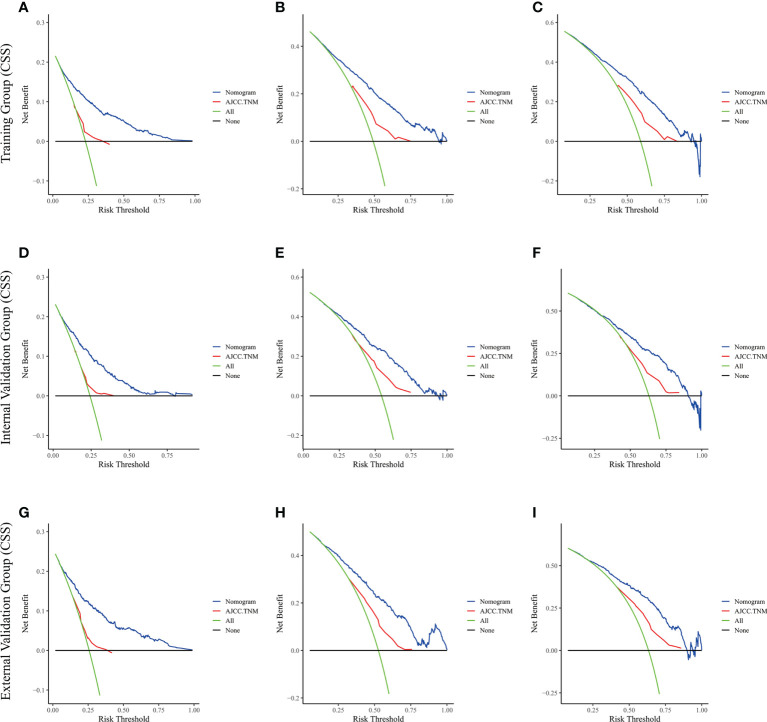
The decision curve analysis of the nomogram and AJCC.TNM for CSS at **(A)** 1-year and **(B)** 3-year and **(C)** 5-year in the training cohort, and at **(D)** 1-year **(E)** 3-year and **(F)** 5-year in the internal validation cohort, and at **(G)** 1-year **(H)** 3-year and **(I)** 5-year in the external validation cohort.

The risk ratios and univariate and multivariate Cox risk models are shown in **Tables 2**, **3**. In the univariate analysis, we identified age, race, marital status, T-stage, N-stage, grade, histological type, surgery, radiotherapy, and chemotherapy as prognostic factors for OS and CSS. Subsequently, after multifactorial analysis of these variables, age (67-80, P<0.001; 81–100, P<0.001), race (Others, P<0.001), marital status (Single, P=0.034; Others, P=0.023), T stage (T3, P<0.001; T4, P<0.001), N stage (N1, P<0.001; N2, P<0.001; N3, P<0.001), grade (II, P<0.001; III, P<0.001), histological type (Special Type, P=0.004), surgery (P<0.001), and chemotherapy (P<0.001) remained statistically significance, indicating significant independent prognostic factors of OS; age (67-80, P= 0.016; 81–100, P=0.018), race (Black, P=0.039; Others, P<0.001), T-stage (T3, P<0.001; T4, P<0.001), N-stage (N1, P<0.001; N2, P< 0.001; N3, P<0.001), grade (II, P=0.001; III, P<0.001), histological type (Special Type, P=0.003), surgery (P<0.001), and chemotherapy (P<0.001) were significant independent prognostic factors of CSS. All variables passed the proportional risk hypothesis test. Independent prognostic factors obtained from univariate and multifactorial regression analyses were then used to construct nomograms of OS and CSS for LAGC patients at 1, 3, and 5 years (**Figure 2**). In the training group, 1-, 3-, and 5-year OS rates were 74.4%, 44.2%, and 33.2%, respectively. The 1-, 3-, and 5-year CSS rates were 77.2%, 49.7%, and 40.8%, respectively. In the internal validation group, 1-, 3-, and 5-year OS rates were 72.0%, 43.5%, and 31.6%, respectively. The 1-, 3-, and 5-year CSS rates were 75.4%, 48.2%, and 37.5%, respectively. In the external validation group, 1-, 3-, and 5-year OS rates were 67.6%, 36.9%, and 24.3%, respectively. The 1-, 3-, and 5-year CSS rates were 69.0%, 40.0%, and 29.0%, respectively. Compared with TNM staging, the nomograms obtained higher C-index values (OS: 0.722[95% CI: 0.708–0.736] in the training cohort; 0.721[95% CI: 0.700–0.742] in the internal validation cohort; 0.728 [95% CI: 0.672–0.784] in the external validation cohort); (CSS: 0.728 [95% CI: 0.713–0.743] in the training cohort; 0.731 [95% CI: 0.709–0.754] in the internal validation cohort; 0.727 [95% CI: 0.668–0.786] in the external validation cohort). The calibration curves showed good agreement between predicted and actual results (**Figures 3**, **4**). ROC assessed the discriminatory ability of the nomogram, and **Figure 5A–C** shows the AUC values for 1-, 3- and 5- OS of nomograms (training cohort: 1-year OS 0.810 [95% CI: 0.787–0.833]; 3-year OS 0.777 [95% CI: 0.756–0.798]; 5-year OS 0.789 [95% CI: 0.767–0.811]; internal validation cohort: 1-year OS 0.804 [95% CI: 0.770–0.838]; 3-year OS 0.781 [95% CI: 0.750–0.812]; 5-year OS 0.764 [95% CI: 0.727–0.802]; external validation cohort: 1-year OS 0.750 [95% CI: 0.652–0.848]; 3-year OS 0.842 [95% CI: 0.769–0.916]; 5-year OS 0.812 [95% CI: 0.723–0.900]); and **Figure 5D–F** shows the AUC values for 1-, 3- and 5-year CSS of Nomogram (training cohort: 1-year CSS 0.808 [95% CI: 0.784–0.832]; 3-year CSS 0.782 [95% CI: 0.761–0.803]; 5-year CSS 0.795 [95% CI: 0.772–0.817]; internal validation cohort: 1-year CSS 0.808 [95% CI: 0.773–0.843]; 3-year CSS 0.786 [95% CI: 0.754–0.818]; 5-year CSS 0.769 [95% CI: 0.731–0.807]; external validation cohort: 1-year CSS 0.741 [95% CI: 0.638–0.845]; 3-year CSS 0.846 [95% CI: 0.772–0.921]; 5-year CSS 0.826 [95% CI: 0.741–0.911]). **Supplementary Tables 1**, **2** indicate that C-index and AUC values of the nomograms are superior to those of TNM staging system. **Figures 6**, **7** compare DCA of each prediction model and AJCC TNM staging. The superior net benefit suggests that the nomogram is more clinically effective than TNM staging.

**Table 2:** Overall Survival Univariate analysis and Multivariate analysis of the training cohort.

Characteristics	Overall Survival Univariate analysis			Overall Survival Multivariate analysis		
	HR	95% CI	P-value	HR	95% CI	P-value
**Age**						
18-66	1	[Reference]		1	[Reference]	
67-80	1.29	1.146-1.453	< 0.001	1.28	1.132-1.447	< 0.001
81-100	2.257	1.943-2.621	< 0.001	1.429	1.209-1.690	< 0.001
**Race**						
White	1	[Reference]		1	[Reference]	
Black	0.845	0.705-1.012	0.068	0.871	0.723-1.050	0.148
Others^1^	0.853	0.746-0.974	0.019	0.796	0.693-0.914	0.001
**Sex**						
Female	1	[Reference]				
Male	1.001	0.894-1.122	0.983			
**Marital status**						
Married	1	[Reference]		1	[Reference]	
Single	1.159	0.993-1.351	0.061	1.188	1.013-1.393	0.034
Others^2^	1.434	1.262-1.631	< 0.001	1.168	1.021-1.335	0.023
**Grade**						
I	1	[Reference]		1	[Reference]	
II	2.323	1.659-3.252	< 0.001	1.973	1.394-2.792	< 0.001
III	2.863	2.069-3.960	< 0.001	2.473	1.766-3.462	< 0.001
IV	1.669	1.019-2.733	0.042	1.648	0.986-2.753	0.057
**AJCC T**						
T1	1	[Reference]		1	[Reference]	
T2	1	0.738-1.356	0.998	1.069	0.787-1.452	0.671
T3	1.365	1.068-1.744	0.013	1.72	1.331-2.224	< 0.001
T4	2.045	1.587-2.634	< 0.001	2.426	1.856-3.172	< 0.001
**AJCC N**						
N0	1	[Reference]		1	[Reference]	
N1	1.148	0.997-1.322	0.056	1.354	1.161-1.581	< 0.001
N2	1.373	1.164-1.619	< 0.001	1.748	1.470-2.078	< 0.001
N3	1.986	1.692-2.331	< 0.001	2.571	2.159-3.062	< 0.001
**Surgery**						
Yes	1	[Reference]		1	[Reference]	
No	2.848	2.528-3.207	< 0.001	3.496	3.070-3.980	< 0.001
**Radiation**						
Yes	1	[Reference]				
No/Unknown	1.133	1.018-1.261	0.022			
**Chemotherapy**						
Yes	1	[Reference]		1	[Reference]	
No/Unknown	1.755	1.558-1.977	<0.001	2.085	1.797-2.420	< 0.001
**Histologic Type**						
Adenocarcinoma	1	[Reference]		1	[Reference]	
Undifferentiated	1.409	0.453-4.377	0.553	1.704	0.509-5.702	0.387
SRCC	1.139	0.990-1.310	0.069	1.008	0.867-1.171	0.919
Special Type	0.739	0.627-0.872	< 0.001	0.772	0.648-0.919	0.004

SRCC, signet ring cell carcinoma; HR, hazard ratio; 95 CI, 95% confidence interval; Others¹, including Asian or Pacific Islander and American Indian/Alaska Native; Others², including separated, divorced and widowed.

**Table 3:** Cancer-specific Survival Univariate analysis and Multivariate analysis of the training cohort.

Characteristics	Cancer-specific Survival Univariate analysis			Cancer-specific Survival Multivariate analysis		
	HR	95% CI	P-value	HR	95% CI	P-value
**Age**						
18-66	1	[Reference]		1	[Reference]	
67-80	1.181	1.037-1.345	0.012	1.180	1.031-1.350	0.016
81-100	1.970	1.666-2.329	< 0.001	1.254	1.040-1.513	0.018
**Race**						
White	1	[Reference]		1	[Reference]	
Black	0.791	0.645-0.970	0.024	0.801	0.649-0.989	0.039
Others^1^	0.800	0.689-0.929	0.003	0.736	0.630-0.859	< 0.001
**Sex**						
Female	1	[Reference]				
Male	1.034	0.911-1.173	0.605			
**Marital status**						
Married	1	[Reference]				
Single	1.158	0.979-1.370	0.087			
Others^2^	1.334	1.156-1.540	< 0.001			
**Grade**						
I	1	[Reference]		1	[Reference]	
II	2.469	1.660-3.674	< 0.001	2.011	1.336-3.026	0.001
III	3.338	2.274-4.899	< 0.001	2.719	1.830-4.040	< 0.001
IV	1.652	0.919-2.971	0.094	1.523	0.819-2.834	0.184
**AJCC T**						
T1	1	[Reference]		1	[Reference]	
T2	1.011	0.716-1.428	0.951	1.076	0.760-1.523	0.681
T3	1.398	1.058-1.847	0.018	1.792	1.340-2.396	< 0.001
T4	2.231	1.676-2.970	< 0.001	2.706	2.001-3.660	< 0.001
**AJCC N**						
N0	1	[Reference]		1	[Reference]	
N1	1.245	1.062-1.460	0.007	1.482	1.247-1.761	< 0.001
N2	1.484	1.234-1.785	< 0.001	1.882	1.552-2.283	< 0.001
N3	2.191	1.835-2.617	< 0.001	2.809	2.316-3.408	< 0.001
**Surgery**						
Yes	1	[Reference]		1	[Reference]	
No	2.964	2.605-3.373	< 0.001	3.768	3.272-4.339	< 0.001
**Radiation**						
Yes	1	[Reference]				
No/Unknown	1.143	1.015-1.286	0.027			
**Chemotherapy**						
Yes	1	[Reference]		1	[Reference]	
No/Unknown	1.653	1.448-1.889	< 0.001	2.074	1.759-2.446	< 0.001
**Histologic Type**						
Adenocarcinoma	1	[Reference]		1	[Reference]	
Undifferentiated	1.665	0.536-5.173	0.378	2.356	0.680-8.158	0.176
SRCC	1.217	1.046-1.415	0.011	1.023	0.870-1.203	0.784
Special Type	0.710	0.589-0.855	< 0.001	0.738	0.606-0.899	0.003

SRCC, signet ring cell carcinoma; HR, hazard ratio; 95 CI, 95% confidence interval; Others¹, including Asian or Pacific Islander and American Indian/Alaska Native; Others², including separated, divorced and widowed.

### Risk stratification

**Figure 8:**
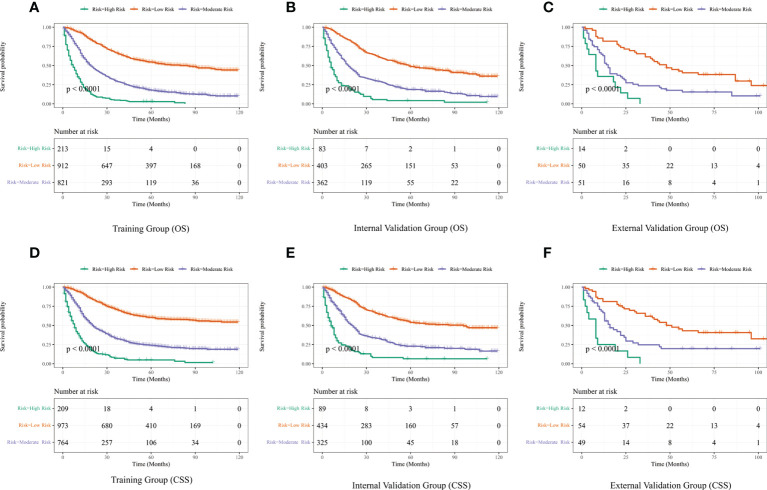
Performance of the nomograms in stratifying on the basis of risk points. **(A)** OS in the subgroups according to the risk stratification in the training cohort. **(B)** OS in the subgroups according to the risk stratification in the internal validation cohort. **(C)** OS in the subgroups according to the risk stratification in the external validation cohort. **(D)** CSS in the subgroups according to the risk stratification in the training cohort. **(E)** CSS in the subgroups according to the risk stratification in the internal validation cohort. **(F)** CSS in the subgroups according to the risk stratification in the external validation cohort.

The best cut-off values were obtained using X-Tile software on the total scores of LAGC patients, which were 210 and 308 for OS and 217 and 309 for CSS. Therefore, LAGC patients were classified into the low-risk group (43–210), the medium-risk group (211–308), and the high-risk group (309–458) for OS. In addition, LAGC patients were classified into the low-risk group (44–217), the medium-risk group (218–309), and the high-risk group (310–449) for CSS. Meanwhile, X-tile classifies patients’ ages into three groups: 18–66, 67–80, and 80–100. **Figure 8** displays the risk stratification of OS and CSS, with survival analysis showing significant differences between these groups (training group OS, p < 0.0001; internal validation group OS, p < 0.0001; external validation group OS, p < 0.0001; training group CSS, p < 0.0001; internal validation group CSS, p < 0.0001; external validation group CSS, p < 0.0001). In the training group, 1-, 3-, and 5-year OS rates were respectively 92.7%, 66.6%, and 54.3% in the low-risk group, 65.9%, 29.6%, and 17.7% in the medium-risk group, and 28.3%, 4.9%, and 2.9% in the high-risk group. The 1-, 3-, and 5-year CSS rates were respectively 93.1%, 70.9%, and 60.4% in the low-risk group, 68.3%, 32.8%, and 23.9% in the medium-risk group, and 33.0%, 7.1%, and 4.9% in the high-risk group. In the internal validation group, 1-, 3-, and 5-year OS rates were respectively 88.8%, 63.0%, and 48.6% in the low-risk group, 63.3%, 30.0%, and 18.7% in the medium-risk group, and 27.5%, 5.5%, and 4.2% in the high-risk group. The 1-, 3-, and 5-year CSS rates were respectively 89.7%, 66.3%, and 53.8% in the low-risk group, 68.6%, 33.5%, and 22.8% in the medium-risk group, and 27.1%, 8.1%, and 6.5% in the high-risk group. In the external validation group, 1-, 3-, and 5-year OS rates were respectively 81.7%, 61.2%, and 40.4% in the low-risk group, 62.7%, 23.5%, and 15.4% in the medium-risk group, and 35.7%, 0%, and 0% in the high-risk group. The 1-, 3-, and 5-year CSS rates were respectively 81.2%, 61.9%, and 43.0% in the low-risk group, 66.5%, 24.6%, and 19.7% in the medium-risk group, and 25.0%, 0%, and 0% in the high-risk group.

### Development of web servers

Based on the nomograms we created, we developed two web servers to predict the OS (https://houchong.shinyapps.io/lagcoverall/) and CSS (https://houchong.shinyapps.io/lagcspecific/) of LAGC patients. By inputting information about LAGC patients, the intertemporal survival probability of patients can be easily predicted, which can better assist clinical efforts.

## Discussion

Due to early gastric cancer patients lacking obvious clinical symptoms, most gastric cancer patients are at locally advanced stages at the time of diagnosis. Currently, clinicians usually evaluate cancer through TNM system, which is generally regarded as the gold standard for diagnosis and treatment and the benchmark for prognosis (25). Given that multiple risk factors other than TNM stage affect the prognosis of cancer patients, TNM staging system ignores the biological heterogeneity of patients, leading to significant differences in prognosis even among patients with the same stage. Therefore, using TNM staging system alone to predict survival is inaccurate. In addition, some studies have found that both new and old versions of TNM staging are not clear in their ability to predict prognosis, and the new version is not more accurate than the old one. This shortcoming is more pronounced in the advanced stages of cancer (26). Therefore, there is a need to develop a more accurate prognostic model to assess the risk of LAGC to provide a convenient and reliable tool for individual survival prediction and develop of treatment strategies for LAGC patients.

The nomogram is based on multifactorial regression analysis, which can predict certain clinical outcomes or the probability of a certain type of event, and its analysis results are graphical and visualized, which can help clinicians make clinical decisions based on the prognosis predicted by the model (21). In this study, first, we comprehensively explored the impact of independent prognostic factors in SEER database on OS and CSS in LAGC patients. Through univariate and multifactorial analyses, we found that age, race, marital status, T-stage, N-stage, grade, histological type, surgery, and chemotherapy were independent prognostic factors for OS in LAGC patients. In addition, age, race, T-stage, N-stage, grade, histological type, surgery, and chemotherapy were independent prognostic factors for CSS in LAGC patients. Second, we developed two nomograms to predict OS and CSS at 1, 3, and 5 years in LAGC patients and found that our models have good predictive accuracy through internal and external validation. Finally, to facilitate clinical use, we set up two web servers based on the nomograms.

When performing prognostic factor analysis, we discovered that elderly patients tend to have a poorer prognosis, which is also true in other cancers. This may be linked to the weaker immune system of the elderly, the weakened reserve function of many of their own organs, and the possibility of comorbidity with multiple diseases. Older patients with gastric cancer are less likely to receive treatment than younger patients because, in contrast to the latter, the former is more likely to have postoperative complications and poor compensatory capacity, eventually leading to poor tolerance of the surgery (27, 28). One study demonstrated that < 20% of elderly patients with LAGC received adjuvant radiotherapy, while in patients aged < 75 years, this percentage was close to 40% (29). After multifactorial Cox regression analysis, sex was not significantly associated with survival in LAGC, consistent with Ji et al.’s study (30). However, in a Korean study, sex was again significantly associated with OS (31). This difference may be attributed to different populations, which must be verified in a multicenter study. Besides, patients who were non-white or non-black had lower mortality rates, consistent with previous studies (32, 33). Our prediction model also presented an association between marital status and OS. A good marital relationship may promote a healthy lifestyle and thus improve survival, and married individuals may also be more proactive in cooperating with treatment because of their spouses’ advice and family responsibilities (34, 35). Traditionally, TNM stage is the most important prognostic indicator for gastric cancer patients. The results of our study are also consistent with this. This is understandable because the deeper the tumor infiltration and the more lymph nodes are involved, the worse the prognosis. In addition, T-stage and N-stage are also key factors for tumor metastasis. The higher the T-stage and N-stage, the greater the risk of tumor invasion into blood vessels or lymphatic vessels for metastasis and the worse the prognosis (36). In this study, the largest number of patients were in grade III, accounting for 65.8%, followed by grade II patients, accounting for 25.6%, and the smallest number was in grade IV, accounting for only 2.7% of the total number of patients. In the nomogram, the risk scores of grade IV patients were lower than those of grades II and III, which may be associated with the small number of grade IV patients and thus may have led to some bias.

Surgery combined with adjuvant chemotherapy is currently an important part of the standardized treatment system for gastric cancer, and aggressive treatment is of great clinical significance in improving the quality and prolonging the survival time of patients (37). Many patients with gastric cancer are at an advanced stage at diagnosis and have never received surgery or chemotherapy before, which may increase the risk of recurrence and death (30). Neoadjuvant chemotherapy may also reduce the staging of LAGC as well as eradicate micrometastases. Neoadjuvant therapy is typically used in patients with clinical staging greater than T2N0 rather than surgery followed by chemotherapy. This approach is more likely to yield better systemic treatment results (38). In contrast, The treatment effect of radiotherapy is not significant. A study from the National Cancer Database indicated that using postoperative radiotherapy decreases due to the increasing use of perioperative chemotherapy. This may be linked to the increased tolerability of perioperative chemotherapy in patients and the significant efficacy achieved with D2 lymph node dissection (39). In histological type, we found that the prognosis of undifferentiated carcinoma was the worst in both OS and CSS, while the prognosis of signet ring cell carcinoma (SRCC) and adenocarcinoma did not differ significantly. This is consistent with the study of Zhao et al., who found little difference in the prognosis of the three types of adenocarcinomas (mixed, classic, and mucinous) and SRCC in advanced gastric cancer (40).

Notably, several significant advantages of our study should be considered. First, this study establish prognostic nomograms for predicting OS and CSS in LAGG patients, and the results of internal and external validation have revealed the reliability and accuracy of nomograms. Second, this study includes other risk factors that affect the prognosis of LAGC patients in addition to TNM stage and classifies patients into three risk groups—high, medium, and low—to provide more information for clinical work. Also, LAGC patients can obtain a total score, find their own risk group according to the nomogram, and then assess their future survival, which has strong clinical guidance. For LAGC patients with high scores and risk, clinicians can make more timely therapeutic interventions and treatment plan adjustments to improve their prognosis. Third, our models can be used to improve the AJCC TNM staging system or as a complementary version. C-index and AUC values of the nomograms for two different study endpoints, OS and CSS, are higher than those of TNM staging in training and two validation groups, indicating that prediction models have better accuracy in predicting patient survival time. The calibration curves also showed a high degree of agreement between predicted and actual observed results, reflecting the reliability of prediction models. Compared with conventional TNM staging system, the clinical benefit of the nomogram was better, as illustrated by DCA curve, which also reflects the superiority of the nomogram. Fourth, based on the nomogram model, we built two network calculators. The ability to predict OS and CSS of LAGC patients more easily and intuitively by inputting patient prognosis-related information provides a convenient tool for clinicians. Finally, Our study can add more useful information to the studies of survival analysis of LAGC. Previous studies have been relatively restrictive regarding the conditions of LAGC patients, such as non-elderly LAGC patients undergoing gastrectomy, LAGC patients over 75 years of age, and patients with locally advanced gastric signet-ring cell carcinoma (29, 41–43). Meanwhile, our study included more treatment modalities, a larger age range, and more histological types. This could provide information for numerous LAGC patients. Additionally, many studies only compared the differences in the effects of different treatment modalities on gastric cancer survival without a predictive model or with only one predictive model (44–47). Meanwhile, our study included more independent prognostic factors and established OS and CSS nomograms.

However, there are some limitations to our study. First, data in SEER database being retrospective information may have led to a potential risk of selection bias during the construction of prediction models. Second, the information in SEER database is incomprehensive, such as the lack of information on cancer patients from other countries and the lack of some other prognostic risk factors associated with survival, such as smoking history, alcohol consumption, *Helicobacter pylori* infection, patients’ body mass index, neurological or vascular or lymphatic vascular invasion, which are also are important factors affecting the prognosis of cancer patients. The absence of such factors may weaken the predictive power of the prognosis of LAGC patients. Finally, the data used to develop and validate the nomogram came only from our hospital and the SEER databases. For example, in our hospital, the patients are only Asian, which inevitably causes some bias in external validation regarding race. Although study results suggest that our nomograms have good predictive accuracy, larger as well as multicenter clinical trials in different countries are required to assess their performance.

## Conclusions

We constructed nomograms that can predict OS and CSS at 1, 3, and 5 years in patients with LAGC and determined the reliability and accuracy of the models through multiple validations. Compared with the AJCC TNM staging, our nomograms performed better in predicting prognosis and can be used as a useful tool to assess the prognosis of LAGC patients, thus contributing to clinical decision-making and individualized treatment planning.

## Data availability statement

The original contributions presented in the study are included in the article/**Supplementary Material**. Further inquiries can be directed to the corresponding author.

## Ethics statement

The studies involving human participants were reviewed and approved by Yantai Affiliated Hospital of Binzhou Medical University Ethics Committee. The ethics committee waived the requirement of written informed consent for participation.

## Author contributions

CH and FY: conception and design. CH and FY: development of methodology. CH: acquisition of data. FY: analysis and interpretation of data. CH and FY: writing, review, and/or revision of the manuscript. CH, FY, and YL: administrative, technical, or material support. CH and FY: study supervision. All authors participated in the writing of the final manuscript and approved the final submission.

## Conflict of interest

The authors declare that the research was conducted in the absence of any commercial or financial relationships that could be construed as a potential conflict of interest.

## Publisher’s note

All claims expressed in this article are solely those of the authors and do not necessarily represent those of their affiliated organizations, or those of the publisher, the editors and the reviewers. Any product that may be evaluated in this article, or claim that may be made by its manufacturer, is not guaranteed or endorsed by the publisher.

## Supplementary material

Supplementary Table 1 C-index for the nomograms and TNM 7th stage in patients with LAGC.

Supplementary Table 2 AUC for the nomograms and TNM 7th stage in patients with LAGC.

Supplementary Table 3 SEER database original data.

Supplementary Table 4 External validation original data.
